# Trauma-sensitive school concepts for students with a refugee background: a review of international studies

**DOI:** 10.3389/fpsyg.2024.1321373

**Published:** 2024-05-02

**Authors:** Eva J. Lembke, Friedrich Linderkamp, Gino Casale

**Affiliations:** School of Education, Institute of Educational Research, University of Wuppertal, Wuppertal, Germany

**Keywords:** trauma-sensitive, trauma-informed, trauma, refugee, school, school-wide

## Abstract

Children and adolescents with a refugee background are at high risk for traumatization. Once they arrive in safe countries, schools are the institutions where teachers are responsible for caring for them sensitively and competently. Furthermore, schools are organized in learning groups consisting of multiple peers of the same age, which provides excellent opportunities for social learning and experiences of social support. In this respect, schools are the appropriate places where preventive concepts can be applied to students with a refugee background. This systematic review summarizes studies that examine or evaluate existing international concepts of trauma-sensitive schools for supporting traumatized students with a refugee background. Based on *N* = 41 selected articles, 17 relevant concepts of trauma-sensitive schools were identified. In 35.3% of the concepts, traumatized students with a refugee background are explicitly included in the target group of the concept, while 47.1% of the concepts refer to groups of students with trauma as a result of various adverse childhood experiences, which also occur more frequently within the population of refugee children and adolescents 17.6% of the concepts contain specific adaptations for pupils with a refugee background. The majority of these concepts were developed in the United States. Additional concepts can be reported for Australia, the United Kingdom, Turkey, and Cambodia. Based on available empirical data, no significant effectiveness regarding the researched concepts’ effects on academic and other school-related data can be determined. Although some studies indicate positive effects concerning school-related target variables, most of the studies have only limited significance due to inadequate research designs and methodological deficiencies. Therefore, there is a great need for further development, careful implementation, and evaluation of trauma-sensitive concepts in schools, especially for the growing group of refugee students.

## 1 Introduction

Globally, 110 million individuals were displaced by the middle of the year 2023. This includes nearly 36.4 million refugees, 6.1 million asylum seekers, and 62.5 million internally displaced persons. In that same year, over 50% of refugees worldwide came from three countries: the Syrian Arab Republic, Afghanistan, and Ukraine. The top five host countries, in descending order, were the Islamic Republic of Iran, Turkey, Germany, Colombia, and Pakistan, while most individual applications for asylum were made in the United States of America, Germany, Spain, Mexico, and France (United Nations High Commissioner for Refugees (UNHCR), 2023). Within Europe, the war in Ukraine has been accompanied by a sharp increase in refugee-related border crossings, with approximately 5.8 million refugees from Ukraine recorded across Europe at the beginning of 2024, while further displacement is to be expected (United Nations High Commissioner for Refugees, 2024a). Overall, 40% of refugees worldwide are under the age of 18 (United Nations High Commissioner for Refugees, 2024b), and thus, in many destination countries, they are also of compulsory school age. Among Ukrainian refugees, the ratio is as high as about 50% (Brücker, 2022).

The health and education systems in the host countries are facing major social challenges due to the high number of refugees and the history of suffering they have already experienced. Furthermore, in addition to insufficient material and human resources, there is a lack of evidence-based trauma-sensitive care and therapy concepts. Schools are particularly confronted with this since trauma-affected students with a refugee background inevitably pass through the education system due to compulsory schooling. Teachers not only have to fulfill the educational mandate but are also confronted with the challenge of supporting traumatized students regarding their individual needs. This requires a deep understanding of trauma-sensitive teaching methods and support interventions.

### 1.1 Forced migration and trauma in childhood and adolescence

Numerous studies have shown that children and adolescents from war zones are at an increased risk of experiencing trauma (Pine et al., 2005; Slone and Mann, 2016; Slone et al., 2017; Khamis, 2019) due to exposure to various traumatic events (Thabet et al., 2006; Khamis, 2019). About one in four children or adolescents experience various fear-inducing situations, such as physical and mental abuse, sexual abuse, domestic violence, accidents, life-threatening illnesses, wars, displacement, death of close relatives, and others (Costello et al., 2002). In the long term, early childhood trauma is a risk factor for a variety of physical and mental illnesses, including heart disease, diabetes, depression, and increased risk behaviors that can lead to other illnesses and social problems. In addition, the risk of suicide is greatly increased (Felitti et al., 1998). Unaccompanied refugee minors are particularly vulnerable to traumatizing experiences as they are largely unprotected in their environment without supporting family members or other adults (Witt et al., 2015). In this regard, Bean et al. (2007) reported a prevalence of physical abuse of about 23% and sexual abuse of 8% among accompanied children with a refugee background in the Netherlands. Among unaccompanied children, physical abuse affected about 63%, and sexual abuse affected about 20%. Multiple and prolonged interpersonal and intentional human-caused traumatic events correlate particularly strongly with psychologically chronic and severely debilitating consequences (Kessler, 1995). Children and adolescents with a refugee background frequently exhibit internalizing and externalizing behavioral concerns as well as symptoms of post-traumatic stress disorder (PTSD) with a prevalence rate of 40–50%, anxiety (about 54%), and depression (32–38%) due to their experiences (Thabet et al., 2006, 2016; Sirin and Rogers-Sirin, 2015; Eruyar et al., 2018; Kandemir et al., 2018; Khamis, 2019; Veale, 2020; Yayan et al., 2020). Common symptoms of PTSD include recurring nightmares, reliving a traumatic experience (flashbacks), sleep disturbances, lack of emotion, anxiety and depression, constant nervousness, and an exaggerated startle response [World Health Organization (WHO), 2019].

Early trauma and changes in the environment caused by flight can also have a long-term negative impact on the psychosocial development of children and adolescents. These changes comprehensively impact all domains of children’s and adolescents’ lives and are attributed to biological, psychological, interpersonal, and contextual dynamics within the framework of biopsychosocial models, which are linked to each other in complex interactions that influence children’s development up to adulthood. At different ages and stages of development, the influence of dynamics in various areas on the psychosocial development and health of individuals is characterized by different weightings (Bronfenbrenner, 1994; Elder, 1994; Halevi et al., 2016; Lehman et al., 2017; Ajrouch et al., 2020).

From a biological perspective, neurophysiological studies have shown that early trauma can adversely affect the brain development of children by impairing brain maturation, overall brain growth, and intelligence development (Bremner and Narayan, 1998; De Bellis et al., 1999; Bremner, 2002; De Bellis et al., 2011). Difficulties often occur in areas of executive function such as working memory, attention, cognitive flexibility, impulse control, and emotion regulation (Perfect et al., 2016; Kavanaugh et al., 2017; Malarbi et al., 2017; Op Den Kelder et al., 2018). Traumatic events can already have an impact on the fetus prenatally through the release of stress hormones, such as cortisol, by the mother, which may be associated with epigenetic changes in the brain and other organs as well as increased sensitivity of the hypothalamic–pituitary–adrenal axis (Carpenter et al., 2017; Huizink and De Rooij, 2018). During early childhood, trauma can also have a negative impact on the developing hypothalamic–pituitary–adrenal axis, causing structural changes in the hippocampus and amygdala, an increase in the number of perceived threats and fear responses, and dysregulation of emotions (Bick and Nelson, 2017). During adolescence, structural changes in the brain, the hypothalamic–pituitary–adrenal axis, and neuronal connectivity make adolescents particularly reactive to environmental influences (Romeo, 2010; Powers and Casey, 2015; Tottenham and Galván, 2016). Impairments in neurophysiological development can be accompanied by changes in children’s behavior that interact with psychological, interpersonal, and contextual dynamics (Lehman et al., 2017; Ajrouch et al., 2020). The extent to which traumatizing life events in the context of flight affect a child or adolescent is, therefore, determined to a large extent by their age and stage of development (Weder and Kaufman, 2011; Siehl et al., 2022).

In infancy and early childhood, parents, especially the mother or other adult caregivers, have a major influence on psychosocial development (Lundberg and Wuermli, 2012; Sangalang et al., 2017; Suárez-Orozco et al., 2018; Zwi et al., 2018; Sim et al., 2019; Goodman et al., 2020; Arakelyan and Ager, 2021; Eltanamly et al., 2021; Gredebäck et al., 2021; Scharpf et al., 2021; Popham et al., 2023). Attachment to caregivers in early childhood plays a critical role in the processing of stressful experiences, as emotion regulation and stress reduction primarily take place in co-regulation with caregivers, whereby children learn long-term skills for self-regulation and affect tolerance (Van Der Kolk, 2006; Feldman and Vengrober, 2011). While the family environment and a good attachment to caregivers with positive parenting styles can, therefore, be an important protective factor (Punamäki et al., 2015; Eltanamly et al., 2021), impairments in parental mental health, such as PTSD, are associated with an unfavorable parenting style that fosters insecure attachment in the child (Eltanamly et al., 2021; Scharpf et al., 2021) and can impair social interaction between parent and child (Gredebäck et al., 2021). This can lead to stress-related changes in parental behavior, resulting in avoidant, overprotective, insensitive, strict, and punitive behavior and even child abuse (Bryant et al., 2018, 2021; Scharpf et al., 2021; Popham et al., 2023), which, in turn, correlates with higher levels of PTSD, depression, and behavioral problems in the children (e. g. Feldman and Vengrober, 2011). A study by Punamäki et al. (2015) shows that children with a refugee background from family dynamics characterized by secure attachment and positive parenting practices have better mental health and can process traumatic experiences more effectively than children from families with insecure attachment and less favorable parenting practices. Parental behavior can, in turn, be significantly influenced by stressful environmental conditions, such as post-migratory stressors (Lundberg and Wuermli, 2012; Bryant et al., 2018; Suárez-Orozco et al., 2018; Sim et al., 2019; Eltanamly et al., 2021; Popham et al., 2023). Using a sample of 1,446 mother–child dyads of Syrian refugee families in Lebanon, Popham et al. (2023) found, based on a holistic model, that the environment of this sample had an impact on the mental health of the child via the mental health of the mother. The age of the child moderates these effects.

Unfavorable attachment patterns in early childhood can also affect the ability to allow relationships with other adult caregivers in later development, for example, when adults are seen as a threat and not as potential help providers (West et al., 2014). In later life, this can also affect the development of relationships with teachers, who can act as supportive, attentive caregivers and provide important support in coping with trauma (Van der Kolk, 2005). This negative effect can be reinforced by teachers who use punitive methods in response to the undesirable trauma-related behavior of refugee students, which in turn can lead to re-traumatization (Hemphill et al., 2014; Howard, 2019). Trauma in infants and young children is more likely to affect the development of internalizing symptoms compared to older children (Kaplow and Widom, 2007; Grasso et al., 2016), while at the same time, it can promote extensive delays in cognitive development, for example, attention span, memory and abstract thinking, problem-solving skills, receptive and expressive language, as well as impairments in inhibitory control, working memory, and executive functions (Cicchetti and Toth, 1995; Cook et al., 2005; DePrince et al., 2009; Shonkoff et al., 2012). This, in turn, can have a negative impact on school performance and learning success (Anda et al., 2006; Miller et al., 2012; Jimenez et al., 2016; Porche et al., 2016).

When children enter school age, school becomes an additional contextual factor for their psychosocial development, as a place where they spend a large part of their time and can establish social contacts with peers, teachers, and other adults outside their family context. Social interactions such as verbal exchange and support in problem situations not only represent an important protective factor to the development of PTSD, but they are also central to the social–emotional development of children and adolescents (Daiute, 2017; Demir et al., 2020; Höltermann et al., 2022). Cohen et al. (2014) find evidence that sharing trauma-related experiences with supportive adults can help adolescents improve their emotional regulation. For children and adolescents with a refugee background, however, social exchange with peers and supportive adults in the school context is considerably more difficult, as they usually do not speak the language of the host country and are not familiar with the cultural context. This not only makes it more difficult to resort to social support as an adaptive coping strategy through peers and advice from teachers, but it also impairs participation in lessons, which can have a negative effect on academic success. Trauma-affected children and adolescents are, therefore, more likely to be rejected by their peers (Schwartz and Proctor, 2000; Boda et al., 2023) and show frequent school performance-related problems such as lower grade point averages and lower graduation rates (Delaney-Black et al., 2002; Terrasi and De Galarce, 2017). On a psychological level, this can impair self-perception and negatively affect the school-related motivation of the children, which can lead to persistent learning deficits in the long term (Lehman et al., 2017). Negative teacher feedback communicated openly in the classroom as a result of poor school performance and behavioral problems caused by emotional dysregulation also carries the risk of having an additional negative impact on social integration (Huber, 2011). Schools, and teachers in particular, therefore, play a central role in the psychosocial development of children in terms of social integration and academic success.

### 1.2 School support for students at risk for trauma

Schools, as highly important and potentially protective environments, have a special responsibility to provide support for students with a refugee background and a risk of traumatization in accordance with their abilities and needs concerning their academic progress as well as trauma-related psychological issues (Kataoka et al., 2018). Teachers and school staff, as the most important trusted adults, can initiate measures to identify and diagnose existing trauma-related symptoms and offer support or refer the child to institutions for additional psychological support. Schools have a crucial function in providing psychological first aid in the context of difficult access to out-of-school therapy due to linguistic, cultural, and bureaucratic barriers. Teachers should, therefore, have a basic knowledge of trauma, its effects on performance, and the social–emotional situation of students in order to recognize and respond appropriately to trauma-related symptoms, support them, and prevent renewed trauma in the school context (Chafouleas et al., 2016; Dorado et al., 2016; Overstreet and Chafouleas, 2016; L’Estrange and Howard, 2022). Social inclusion and support, as well as emotional regulation, are proven protective factors against the development of PTSD (Demir et al., 2020; Höltermann et al., 2022). According to a survey of 304 classes from German schools, students with refugee experience, in particular, have fewer friends than their classmates and are rejected more frequently, although this effect was less pronounced in classes with a highly heterogeneous student body (Boda et al., 2023). Friendships do not only offer social support to students with refugee experience. Social contacts with the majority group in particular offer students with a refugee background important resources for acquiring the language of the host country, thus increasing their chances in the education system and the labor market and acculturating overall (Edele et al., 2020; Lorenz et al., 2021). Supporting social integration is therefore not only of individual importance for the development of the students concerned but is also of long-term interest concerning current political discourses on migration and inclusion policy (Lorenz et al., 2021; Boda et al., 2023; Organisation for Economic Co-operation and Development, 2023). While trauma-sensitive school concepts are increasingly being established in the United States of America (Simon et al., 2020), limited efforts have been made to implement such concepts into European school systems. Teachers often lack a comprehensive understanding of the neurophysiological, psychological, academic, and behavioral effects of trauma on their students. This hinders their ability to recognize and appropriately respond to symptoms of trauma. Moreover, they are often insufficiently trained in school-based strategies for supporting students who have experienced trauma.

### 1.3 Trauma-sensitive schools

Due to its human, material, spatial, and social resources, the school has the necessary prerequisites to carry out preventive measures to support students with a refugee background in the event of traumatization, in addition to specific interventions in the event of trauma (Ellis et al., 2013). Trauma-sensitive concepts are organized holistically and, in addition to helping people cope with trauma-related symptoms, consider aspects such as self-regulation, well-being, physical and emotional health, and academic competence (Cole et al., 2005, 2013). Developing a trauma-sensitive school requires processes of change at all levels of schools, including the way they run, trauma-sensitive adaptation of all school policies and guidelines, their spatial design, and the use of evidence-based testing and support measures for affected students. Additionally, collaborating with external organizations and involving parents and other key caregivers of students is vital (Cole et al., 2005, 2013; Substance Abuse and Mental Health Services Administration (SAMHSA), 2014; Chafouleas et al., 2016).

The first comprehensive approach in the United States exclusively related to the development of trauma-sensitive schools, *Helping Traumatized Children Learn – A Report and Policy Agenda* (Cole et al., 2005) was published by the *Trauma and Learning Policy Initiative (TLPI)* and later expanded in 2013 with a second volume, *Helping Traumatized Children Learn – Creating and Advocating for Trauma-Sensitive Schools* (Cole et al., 2013), which provided guidance and further recommended actions for designing trauma-sensitive schools. This flexible framework for designing trauma-sensitive learning environments in schools includes guidance for transformations in the domains of (1) leadership, (2) professional development, (3) access to resources and service delivery, (4) in-school and out-of-school strategies, (5) policies and regulations, and (6) collaboration with families.

In addition, the following specific characteristics of trauma-sensitive schools are outlined:

- Leadership and staff share an understanding of trauma’s impacts on learning and the need for a school-wide approach.
- The school supports all students to feel safe physically, socially, emotionally, and academically.
- The school addresses students’ needs in holistic ways, taking into account their relationships, self-regulation, academic competence, and physical and emotional well-being.
- The school explicitly connects students to the school community and provides multiple opportunities to practice newly developing skills.
- The school embraces teamwork, and staff share responsibility for all students.
- Leadership and staff anticipate and adapt to the ever-changing needs of students (Cole et al., 2013: 18)

Another trauma-informed care (TIC) concept that has been adapted for the school context and has influenced many of the subsequent trauma-sensitive school concepts is the Substance *Concept of Trauma and Guidance for a Trauma-Informed Approach*:

*A program, organization, or system that is trauma-informed realizes the widespread impact of trauma and understands potential paths for recovery; recognizes the signs and symptoms of trauma in clients, families, staff, and others involved with the system; and responds by fully integrating knowledge about trauma into policies, procedures, and practices, and seeks to actively resist re-traumatization* (Substance Abuse and Mental Health Services Administration (SAMHSA), 2014: 9).

Key principles include trauma-sensitive adaptations in terms of *Safety*, *Trustworthiness, Transparency*, *Peer Support*, *Collaboration and Mutuality*, *Empowerment, Voice and Choice,* and *Cultural, Historical, and Gender Issues.* These principles must be realized in ten implementation areas: *Governance and Leadership*, *Policy*, *Physical Environment*, *Engagement and Involvement*, *Cross Sector Collaboration*, *Screening, Assessment and Treatment Services*, *Training and Workforce Development*, *Process Monitoring and Quality Assurance*, *Financing*, and *Evaluation*.

Chafouleas et al. (2016) integrated the key principles and implementation domains established by SAMHSA into a multi-tiered diagnostic and support concept (Multi-Tiered System of Support; MTSS) and transformed it into a blueprint for implementing trauma-informed approaches in schools. MTSS is usually organized on three successive levels (tiers) with increasing intensity of diagnostic and support approaches. Assignment of students to the respective tiers is done preventively through regular data collection at each tier (Grosche and Volpe, 2013; Simon et al., 2020; Linderkamp and Casale, 2023) and without the need for stigmatization (by, e.g., identifying support needs). MTSS has been mandated by law since 2001 through the *No Child Left Behind Act* and has subsequently been implemented in numerous schools throughout the United States (Reinbergs and Fefer, 2018; Simon et al., 2020).

Tier 1 includes school-wide and universal strategies directed toward all students. These strategies promote a positive school climate, reduce negative conditions, and enhance social problem-solving and coping skills. They may be combined with established approaches, like School Wide Positive Behavior Support or Social–Emotional Learning. Tier 2 provides additional support to students identified as needing increased assistance or who are at a higher risk of experiencing trauma due to the Tier 1 diagnostic process. To assist these students, Tier 2 employs various approaches, such as psychoeducation related to trauma, strengthening social support systems, and improving self-regulation skills. Typically, this support is offered in small groups within the school setting. Tier 3 entails conducting intensive and specific interventions to mitigate trauma-related symptoms, frequently utilizing approaches such as cognitive behavioral therapy (CBT) (Chafouleas et al., 2016; Berger, 2019; Linderkamp and Casale, 2023). CBT-based interventions achieve moderate to large effects in school contexts in terms of reducing PTSD symptoms (Rolfsnes and Idsoe, 2011).

The intervention most commonly integrated into three-tiered trauma-sensitive school concepts is the Cognitive behavioral intervention for trauma in schools (CBITS; Jaycox, 2003). Further notable interventions based on CBT include the Support for Students Exposed to Trauma (SSET; Jaycox et al., 2009) and the specialized approach of trauma-focused cognitive–behavioral therapy (Hansel et al., 2010; Farina et al., 2018). Some U.S. concepts explicitly refer to close collaboration with mental health services in order to assist students in accessing trauma-specific therapy when it is not available through the school itself (Chafouleas et al., 2016; National Child Traumatic Stress Network Schools Committee (NCTSN), 2017). Four-tiered models may involve parental and community engagement (Ellis et al., 2013). Despite major overlaps in terms of content, the final implementations within the respective tiers might vary (Berger, 2019). Four-tiered models may involve parental and community engagement (e.g., Ellis et al., 2013).

According to Maynard et al. (2019), three criteria were created to facilitate the identification of trauma-sensitive whole-school concepts as follows:

1. Workforce/PD components of the program are designed to increase the knowledge and awareness of school staff on the impact, signs, and symptoms of trauma, including secondary traumatization. PD does not necessarily have to be provided to all school staff in a school, but there must be some staff development component as part of the program.
2. Organizational change may include school-wide policies and procedures and/or strategies or practices intended to create a trauma-informed environment integrating the key principles of the trauma-informed approach.
3. The concept must implement changes in practice behaviors across the school, including trauma-specific screening, prevention, and/or intervention services (Maynard et al., 2019: 9).

Reviews of trauma-sensitive school concepts cover various areas of the school environment and yield divergent conclusions based on the underlying research question (Berger, 2019; Thomas et al., 2019; Fondren et al., 2020; Stratford et al., 2020; Avery et al., 2021; Cohen and Barron, 2021; Roseby and Gascoigne, 2021).

Stratford et al. (2020) developed a taxonomy of techniques for ensuring trauma-sensitive practices within schools. The system includes *policies* (guidelines for addressing trauma), *programs* (structured activities designed to address trauma), and *practices* (actions or series of actions aimed at addressing trauma). Components can vary in their dosages, approaches (*Universal, Selected, Targeted, Sequenced*), and objectives (such as identification, referral, promotion of coping strategies, or the creation of a positive classroom climate) across different levels of the school, classroom, and outside the classroom.

Maynard et al. (2019) conducted a comprehensive review of the literature on the impact of trauma-informed approaches in schools. Based on their definition of trauma-informed school concepts, the researchers were unable to find any studies with a randomized or quasi-experimental design with comparison groups in a school setting (PreK–12 or similar) that examined the effects of trauma symptoms/mental health, academic performance, behavior, or socioemotional functioning at the student level.

In a study conducted in the same year, Berger (2019) identified a total of ten three-tiered and three 4-tiered concepts for TIC in schools. In a review of the effect of trauma-informed educational programs on the academic achievement of students who were exposed to adverse childhood experiences (ACEs) in childhood, Roseby and Gascoigne (2021) identified 15 programs that (a) were implemented at the whole-school level, (b) targeted participants who were directly or indirectly affected by ACEs, and (c) examined a school performance-related effect like grades, attendance, academic performance, standardized performance, or discipline as the dependent variable. Existing concepts for adapting to refugee students’ backgrounds were not analyzed, even though they experience higher trauma rates. Avery et al. (2021) provided an overview of school-wide trauma-informed approaches that required at least two of the following characteristics to be met, following the trauma-sensitive school characteristics of Substance Abuse and Mental Health Services Administration (SAMHSA) (2014) and TLPI (Cole et al., 2005, 2013) “(1) staff professional development directly related to understanding the impact of trauma (2) Practice change – implement changes in practice behaviors across the school i. e.: trauma screening, prevention and/or intervention and an intentionality toward relational connection with students and (3) Organizational change – includes policies and procedures, strategies or practices to create a trauma-informed environment i.e.: policy relating to disciplinary practices” (Cole et al., 2013: 383). Studies that were limited solely to an evaluation of effects using trauma screening, assessment, or treatment of trauma symptoms were excluded. In this process, four scholarly articles relating to four school-wide concepts were identified: *Healthy Environments and Response to Trauma in Schools* (HEARTS; Dorado et al., 2016), *The Heart of Teaching and Learning: Compassion, Resiliency, and Academic Success* (HTL; Day et al., 2015), *The New Haven Trauma Coalition* (NHTC; Perry and Daniels, 2016), and *Trust-Based Relational Intervention* (TBRI; Parris et al., 2015). Overall, trauma-informed programs have been shown to improve academic performance in schools, although studies have produced varying results depending on the specific variables and outcomes examined. The impact of trauma-sensitive school concepts on students with a refugee background who have experienced trauma is not established. However, the importance of further research in this area is emphasized by all reviewed articles.

Despite the high relevance given the global political situation and the growing number of refugee children and adolescents attending schools in different countries, no research has yet focused on trauma-sensitive concepts that support this particular group or explicitly address them in their design. The purpose of this article is to provide a systematic review of international studies on concepts of trauma-sensitive schools that aim to support traumatized students with a refugee background. Due to the very dynamic developments in global refugee movements in recent times, the currency of such studies is of particular importance here. The research is based on the following research questions:

1. What concepts of trauma-sensitive schools exist internationally that address the group of traumatized students with a refugee background?
2. Which adaptations do the concepts include for refugee students who have experienced trauma?
3. How are the concepts distributed worldwide in terms of their conception?
4. What empirical evidence is available regarding the impact on academic and school-related aspects of concepts of trauma-sensitive schools that address the group of traumatized students with a refugee background?

## 2 Materials and methods

The study examines the research question based on a comprehensive database literature search regarding existing concepts of trauma-sensitive schools worldwide.

### 2.1 Literature search

The following platforms and databases were screened during the literature search:

- EBSCOHost (MEDLINE, Psychology and Behavioral Sciences Collection, APA PsycARTICLES, APA PsycINFO, Psyndex Literature, EBSCO eBook Collection, OpenDissertations)
- ProQuest (ERIC, PTSDpubs, Social Services Abstract, Sociological Abstract)
- FIS Bildung
- PubMed
- Database of the University of Wuppertal
- Google Scholar

The keywords used for the literature search were generated from the current English-language literature on trauma-informed research in schools (Carter and Blanch, 2019): (trauma-informed OR “trauma informed” OR trauma-sensitive OR “trauma sensitive” OR trauma-responsive OR “trauma responsive” OR trauma-aware OR “trauma aware”) AND school AND (refuge* OR asyl* OR).

**Figure 1:**
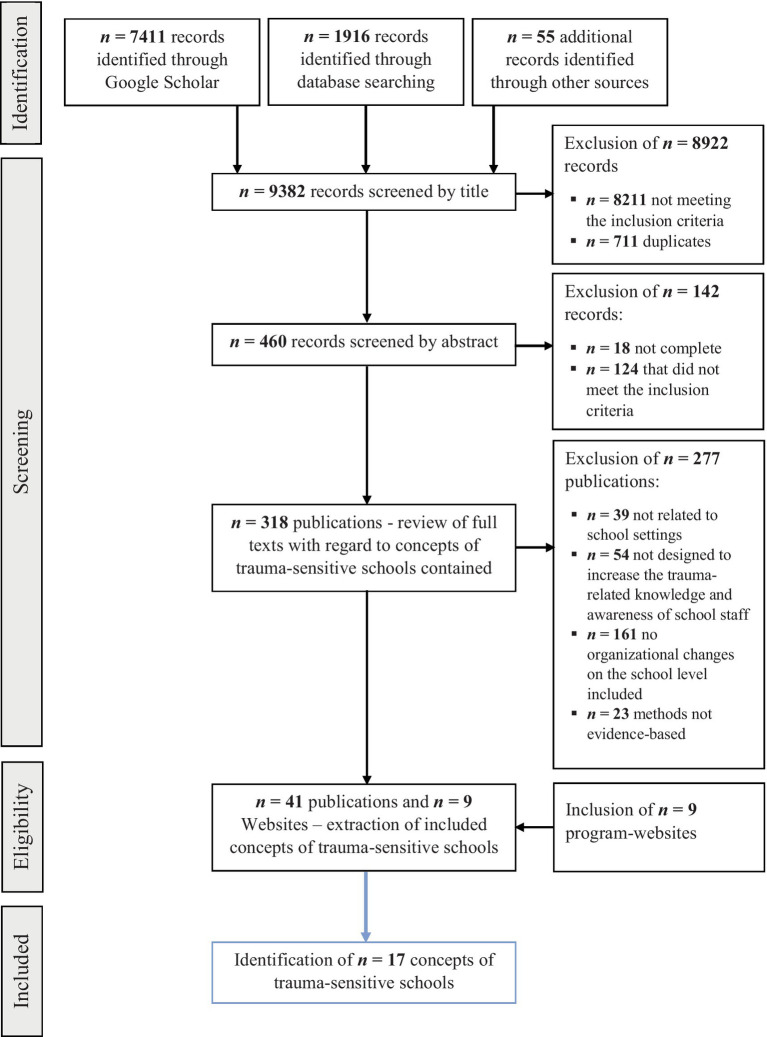
Flowchart describing the process and results of the literature review [adapted from Moher et al. (2009)]. The area highlighted describes the process of extracting concepts of trauma-sensitive schools from the included documents.

Additional records were identified through the websites of journals, the U.S. Department of Education, the NCTSN, independent trauma-sensitive school concepts, and bibliographies (see Figure 1). The research and selection were conducted by a single person. The search via Google Scholar revealed a saturation of results after approximately 500 search results, so the remaining results were roughly screened according to this number using the titles.

### 2.2 Selection strategy

The understanding of trauma-sensitive school concepts that underlie this study is based on the core tasks of trauma-sensitive systems articulated by Substance Abuse and Mental Health Services Administration (SAMHSA) (2014). Based on this, the criteria formulated by Maynard et al. (2019) were used to identify trauma-sensitive school concepts:

1. The trauma-sensitive school concept is designed to increase the knowledge and awareness of school staff (and groups thereof, as appropriate) about the signs and symptoms of trauma, its effects, and the importance of trauma-sensitive approaches in schools.
2. The concept includes organizational changes, such as school-wide policies or practices to develop a trauma-sensitive environment.
3. The concept involves a change in practice that includes an application of evidence-based methods for dealing with trauma.

In this study, concepts were considered in which the three criteria described were met. Following Stratford et al. (2020), publications that provide theoretical guidelines and guidance for implementing trauma-sensitive school concepts (“policies”), as well as programs and studies with an underlying approach that is or has been implemented in practice, are included in the data extraction process. Due to a lack of relevance to our research question, concepts relating to kindergartens or preschools were not included.

The selection process followed the PRISMA guidelines for systematic review and meta-analysis (Moher et al., 2009). Within the literature search using the above-mentioned databases, a total of 9,363 hits were recorded during the survey period of October 13–21, 2021 and August 20–27, 2023 (including 7,411 hits in Google Scholar). Due to the high number, the selection was limited to the first 500 results displayed (sorted by relevance). The selection of relevant articles and other literature can be seen in Figure 1. A total of 460 documents were included in the abstract analysis. This was followed by a review of the available documents regarding further concepts of trauma-sensitive schools and the inclusion of additional literature. A total of 41 publications were identified that were relevant to answering the research question.

Quantitative, qualitative, and mixed-methods studies were reviewed to evaluate the effectiveness of trauma-sensitive school concepts. The non-randomized design was used in the quantitative and mixed-methods studies, and in many cases, there was no control group. In most studies, multiple interventions were evaluated. The risk of bias was therefore examined using the *Risk Of Bias In Non-randomized Studies of Interventions tool* (ROBINS-I; Sterne et al., 2016). The risk of bias is assessed in the domains (a) bias due to confounding, (b) bias in the selection of participants into the study, (c) bias in classification of interventions, (d) bias due to deviations from intended interventions, (e) bias due to missing data, (f) bias in the measurement of outcomes, and (g) bias in the selection of the reported result and both at the domain level and at the overall level with “low,” “moderate,” “serious,” “critical” risk of bias, or “no Information.” Due to a lack of adequate instruments to calculate the risk of bias in mixed-methods approaches for non-randomized intervention studies, the quantitative elements of the studies were also analyzed separately with the ROBINS-I and the qualitative study parts. The majority of the studies examined showed an increased risk (“critical” or “serious”), while only two studies showed a “low” risk of bias.

Qualitative studies and study elements were evaluated using the *CASP Qualitative Studies Checklist* (Critical Appraisal Skills and Programme, 2018). The checklist focuses primarily on the quality assessment of qualitative research but also includes two items that consider the assessment of bias risk in the areas of (a) researcher bias and influence during the formulation of research questions, data collection, including recruitment and site selection, and during (b) analysis and selection of data for presentation. The majority of the studies did not contain sufficient information to allow a well-founded analysis of the risk of bias about these criteria. Therefore, to assess the effectiveness of trauma-sensitive school concepts, studies were not excluded due to an increased risk of bias.

### 2.3 Data extraction

For data extraction, the reference of the documents, the respective country, the type of document, if applicable, the internet presence, and the respective title of the concept contained within were documented (Table 1). Since the literature partly shows overlaps considering the underlying concepts, several references for the respective concepts were noted in these cases.

**Table 1:** Summary of trauma-sensitive school concepts worldwide.

Concept name (acronym)	Publication(s), main reference	Country	Concept type	Target group	Concept description	Implementation realized by
***Arora et al. (2021): “A three-tiered model for addressing the mental health need of immigrant-origin youth in school”	Arora et al. (2021)	USA	Policy, MTSS	Potentially traumatized students with a migration background in adolescence (immigrant-origin youth)	Concept for promoting the mental health of immigrant youth and their families with suggestions for implementation in school practice based on empirical findings. Three levels: (1) Universal, supportive strategies that benefit immigrant students and are school-wide (e.g., SEL, Resilience Classroom Curriculum, strategies to improve classroom climate, family involvement interventions) (2) Selective and specialized support, some of which is group-based (e.g., Mental Health Literacy Program, culturally sensitive programs for immigrant youth and their families). (3) Intensive, some individualized (e.g., TF-CBT, CBITS) support at Levels 2 and 3 can occur inside or outside of school, depending on resources.	School staff
**Berry Street Education Model	Brunzell et al. (2015a), Farrelly et al. (2019), Stokes et al. (2019), Stokes and Turnbull (2016), and Berry Street (2023)	Australia	Program	Traumatized students (non-specific/ACEs), all grades	Concept that implements the framework of TIPE in the form of an alternative educational approach in schools. More than 100 combinable strategies that teachers can draw on as part of the concept’s curriculum relate to five domains: (1) Body—building students’ skills: Inside by improving physical regulation of stress response, de-escalation, and concentration, (2) Relationship—promoting task-based learning through relationship-based classroom management strategies, (3) Perseverance—creating a culture of academic perseverance by promoting resilience, emotional intelligence, and a growth mindset, (4) Engagement—strategies that increase readiness for learning, (5) Utilizing Values and Character Strengths. Training and mentoring by program staff are an integral part of the program.	School staff
* Chafouleas et al. (2016): “Toward a blueprint for trauma-informed service delivery in schools”	Chafouleas et al. (2016), Chafouleas et al. (2019), and Kataoka et al. (2018)	USA	Policy, MTSS	Traumatized students (non-specific/ACEs), all grades	Concept based on the guidelines of trauma-informed organizations (Substance Abuse and Mental Health Services Administration (SAMHSA), 2014), applied to schools for the first time as a blueprint. Three levels: (1) universal (e.g., positive school climate, reducing negative environmental conditions, promoting problem-solving and coping skills, teaching behavioral expectations), (2) targeted (e.g., trauma-informed psychoeducation, promoting social support systems, strengthening self-regulation skills), (3) Selective (psychological interventions to reduce the impact of trauma and re-traumatization, e.g., CBT or referral to psychotherapeutic service providers).	School staff, cooperating instances
*Collaborative Learning for Educational Achievement and Resilience (CLEAR)	Blodgett (2019), Blodgett and Dorado (2016), Washington State University (2016), Washington State University (2018), and Washington State University (2023)	USA	Program, MTSS	Traumatized students (non-specific/ACEs), all grades	Concept that focuses specifically on the use of evidence-based trauma-sensitive practices that are trained with guidance from program staff and combined with trauma-sensitive language. The goal is, after an implementation period of three years, to develop basic strategies, decision-making structures, leadership practices, and skills of educators to the point where trauma-sensitive practices are self-sustaining. CLEAR may or may not be implemented in a multi-tiered system. Training and guidance by program staff is an inherent part of the concept.	School staff, cooperating instances
**Compassionate schools/The Heart of Teaching and Learning (HTL): Compassion, resiliency, and academic success	Day et al. (2015), Wolpow et al. (2009)	USA	Program	Traumatized students (non-specific/ACEs), all grades	Concept that emphasizes the promotion of resilience in students and creation of a co-leadership environment that incorporates and explicitly addresses trauma-sensitive approaches. Drawing on research, ecological and educational theories, and psychoeducational cognitive-behavioral and relational approaches, the concept contains a curriculum that can be used in a variety of educational settings. The Heart of Learning and Teaching: Compassion, Resiliency, and Academic Success (Wolpow et al., 2009) handbook provides extensive recommendations for implementation related to instructional principles, curriculum areas, strategies, teacher self-care, and school-community partnerships. In-service Training is provided.	School staff, parents, cooperating instances, employees of the project
*Hagar-Model:	Wyatt et al. (2017) and Wyatt et al. (2018)	Cambodia	Program	Traumatized students (non-specific/ACEs), all grades	Concept whose description of structure and content are part of recent empirical research (Wyatt et al., 2017, 2018). In a qualitative survey with 14 teachers at one school, the core strategies identified were encouragement and empowerment, behavior management strategies, collaboration, fostering relationships and coping with trauma. Teacher training is an integral part of the program.	School staff, employees of the project
*Healthy Environment and Response to Trauma in Schools (HEARTS):	Blodgett and Dorado (2016) and Dorado et al. (2016)	USA	Program, MTSS	Traumatized students (non-specific/ACEs), all grades	Concept designed to reduce the amount of time spent in the classroom on disciplinary measures and thus increase effective instructional time. Three levels: (1) primary intervention (80% of students; building capacity of school staff, e.g., trauma-informed training and self-care for staff, using a trauma-sensitive perspective to strengthen universal support, e.g., school climate support, PBIS, SEL, restorative justice), (2) early secondary intervention (for 15% of students; e.g., team meetings for at-risk students, trauma-informed training and self-care for staff, using a trauma-sensitive perspective to strengthen universal support, e.g., school climate support, PBIS, SEL, restorative justice). For example, team meetings for at-risk students, trauma-sensitive, social justice, and anti-racist behavior support systems, (3) Intensive, tertiary intervention (for 5% of students; trauma-specific psychotherapy for students, trauma-sensitive crisis management, and consultation with teachers by program staff). Training is offered to school staff and cooperation partners, workshops for parents, support and counseling for teachers, and optional individual psychotherapy for traumatized students by a program staff member on several days at the school.	School staff, parents, cooperating instances, employees of the project
**Helping Traumatized Children Learn (HTCL)	Atallah et al. (2019), Jones et al. (2018), Cole et al. (2005, 2013), and Trauma and Learning Policy Initiative (TLPI) (2023)	USA	Policy	Traumatized students (non-specific/ACEs), all grades	“Flexible Framework,” two manuals with comprehensive recommendations for schools to implement measures to move toward a trauma-sensitive school in the areas of school mobilization, leadership, the development of action plans, and educational support strategies. The second volume additionally contains far-reaching suggestions for educational policy changes related to trauma sensitivity.	School staff, cooperating instances
*Missouri Model:	Alive and Well Community (2019) and Carter and Blanch (2019)	USA	Policy	Traumatized students (non-specific/ACEs), all grades	Concept in which the development of a trauma-informed school is understood as a process that is operationalized based in of various indicators at different levels in different domains. Depending on these indicators, schools can be assigned to the levels “Pre-Trauma Aware,” “Trauma Aware,” “Trauma Sensitive,” “Trauma Responsive,” and “Trauma Informed.” Different domains each display different levels of progress in the development process. The indicators can be used as targets for reaching the next level. In addition, there is a range of training courses.	School staff
**National Child Traumatic Stress Network Schools Committee (NCTSN) (2017)	National Child Traumatic Stress Network Schools Committee (NCTSN) (2017)	USA	Policy, MTSS	Traumatized students (non-specific/ACEs), all grades	Concept with ten key areas of trauma-sensitive schools, which are organized according to the different tiers and contain instructions for action. Three levels: (1) Universal (building and supporting a trauma-sensitive school community and safe environment that benefits all students, e.g., improving school climate, emergency management, bullying prevention), (2) Early intervention and identification of at-risk students (e.g., including forms of CBT and peer support), and (3) Intensive support (e.g., through individual and/or family therapy and trauma-specific treatment). Specific key strategies and key partnerships are formulated for each stage.	School staff, cooperating instances
*Rethinking Learning and Teaching Environments (ReLATE)	Diggins (2021)	Australia	Program	Traumatized students (unspecific/ACEs) at a specialist school for students with learning needs or social and/or emotional challenges	Concept that synthesizes based on multiple concepts (see right column of table) school-wide trauma-specific interventions that include a correction of dysregulated stress responses, the enhancement of self-regulation skills, embedding routines and rituals for the purpose of establishing safety and predictability, and building relationship skills.	School staff
***School’s In for Refugees	Grant and Francis (2011) and Foundation House (2023)	Australia	Program	Traumatized students (non-specific/ACEs), all grades	Concept, which is carried out in cooperation with the Department of Education and Training Victoria, among others, and is financially supported by the latter. Schools can participate in the program free of charge. At the heart of the concept is the Refugee Education Support Program, which provides teachers with basic knowledge about refugee-related trauma, its impact on learning, and classroom-based strategies for dealing with students with a refugee background and trauma. Staff from the organization use the materials and network partnerships to create customized programs for schools that include action plans, resource provision, professional development, and promotion of collaboration with parents. The materials address teaching and learning, school climate, transitions, families and partnerships, and professional leadership, and draw on scientific evidence.	School staff, employees of the project
**The Sanctuary Model	Banks and Vargas (2009), Bloom (2007), Bloom (2014), Esaki et al. (2013), Matey (2014), National Child Traumatic Stress Network (NCTSN) (2008), Yanosy et al. (2015), and Andrus Sanctuary Institute (2023)	USA	Policy	Traumatized students (non-specific/ACEs), all grades	Concept was originally developed as an evidence-based intervention within mental health services and adapted in various schools within the USA. At its core, a change process is built on three components: 1. theoretical principles, 2. a common trauma-sensitive language (S.E.L.F.), 3. tools for practical implementation (Santcuary Tool Kit). Training offered; implementation in schools also in the United Kingdom and Northern Ireland as well as Australia.	School staff, employees of the project
***Trauma informed schools	Maya Vakfı (2019) and Maya Vakfı Foundation (2023)	Türkey	Program, MTSS	Traumatized students (non-specific/ACEs), all grades	Concept that primarily addresses the target group of Syrian refugee students with trauma. Three steps: (1) Establishment of a safe environment from which all students benefit, (2) Screening for trauma-related symptoms and intervention in small groups by the Maya Vakfı Foundation, (3) Measures to build resilience and reduce trauma-related symptoms in the field office of the Maya Vakfı Foundation. Within the framework of a training course, teachers are trained in the knowledge of trauma, its effects, trauma in connection with displacement and the frequently correlated causes of trauma, as well as strategies for dealing with traumatized students at school with regard to various strategies.	Teachers, school administrators and school counselors, Maya Vakfı
*Trauma Informed Schools UK (TISUK)	Demkowicz and Humphrey (2019) and Trauma Informed Schools UK (2023)	United Kingdom	Program	Traumatized students (non-specific/ACEs), all grades	Concept that is being implemented in schools across the UK and internationally. The non-profit organization behind it offers training for individuals on trauma and mental health at different levels of intensity, training for whole schools (with the option of implementing a whole-school approach), and training for student counseling and webinars.	School staff, employees of the program
*Trauma-Informed Positive Education (TIPE)	Stokes and Brunzell (2019), Brunzell et al. (2016), and Brunzell et al. (2015b)	Australia	Policy	Traumatized students (non-specific/ACEs), all grades	Concept was implemented across the United Kingdom and internationally in schools. The non-profit organization behind it provides training for individuals on trauma and mental health at various levels of intensity, training for whole schools (with the option of implementing a whole-school approach), and training for counseling students and webinars.	School staff
**Trauma-Sensitive School Training Package (TSSTP)	Guarino and Chagnon (2018), Delaney (2020), and National Center on Safe Supportive Learning Environments (2023)	USA/ United Kingdom	Program, MTSS	Traumatized students (non-specific/ACEs), all grades	Concept, which provides a basis for various implementation guides, informational materials for understanding trauma, as well as guidance for building and managing trauma-sensitive schools and supplementary materials (including reflection materials). Three levels: (1) School-wide strategies (relate to trauma and resilience building and are preventive and proactive to all students), (2) Secondary interventions (group interventions for students at risk), (3) Tertiary, individualized interventions. Offering training and webinars	School staff, cooperating instances

The relation of the respective concepts to the group of traumatized students with a refugee background is divided into three groups based on the asterisks: ***the concept includes measures for the group of traumatized students with a refugee background. **the concept explicitly considers the group of traumatized students with a refugee background, and *the concept refers to trauma-related experiences that may occur among the group of students with a refugee background, but does not explicitly list them as part of the target group. In the context of this work, the term “school staff” is understood to mean the entire staff of a school in pedagogical, nursing, medical, managerial, or supportive positions, including the school management, teachers, school psychologists, inclusion assistants, social workers, and other staff.

For an overview of the concepts, all documents referring to the same concept were compared, and the document in which the concept was described for the first time was listed as the main reference. In the first descriptive step, the concepts were classified into policies and programs according to their practical relevance. In addition, subgroups that are primarily addressed by the concept or the trauma-specific measures contained therein were identified, and the respective implementation approaches were elaborated. In addition, the concepts were classified with regard to their relation to the subgroup of traumatized students with a refugee background. A distinction was made between three types of reference:

1. Concepts that directly address traumatized students with a refugee background and/or contain specific measures for this group.
2. Concepts that explicitly consider traumatized students with a refugee background and report this in a written form.
3. Concepts that refer to trauma-related experiences that can occur in the context of forced migration, but do not explicitly mention students with a refugee background as part of the target group.

To assess the effectiveness of trauma-sensitive school concepts, empirical publications that examined the impact on various outcomes were summarized in terms of the following characteristics:

- Status of publication in a journal with the peer-review process
- Setting of the concept
- Size and composition of the sample
- Evaluation design
- Existence of a description of the concepts’ implementation by school staff
- Length of intervention (in most cases, time elapsed between implementation of the concept in a school and the survey)
- Dependent variables, which are classified in terms of their target group into variables related to school staff, school, and class level, or students
- Summary of results

The concepts were then compared according to these dimensions and discussed in terms of their significance and comparability.

## 3 Results

A total of 41 documents and nine websites were identified, which are summarized and presented as an overview in Table 1. Most documents are journal articles describing concepts of trauma-sensitive schools descriptively (24%) or empirically (26%). Other document types include websites of trauma-sensitive school concepts (18%), informational and training materials (12%), project reports (12%), and monographs/manuals (6%). Additionally, one dissertation (2%) was included in the evaluation.

### 3.1 International concepts of trauma-sensitive schools

Based on the documents and websites listed, 17 concepts of trauma-sensitive schools were identified that met the inclusion criteria. 58.8% of these concepts were developed in the USA. Other trauma-sensitive school concepts originate from Australia (23.5%), the United Kingdom, Turkey, and Cambodia (5.9% each). In 58.8% of the cases, the programs are linked to at least one training intervention (Wolpow et al., 2009; Guarino and Chagnon, 2018; Maya Vakfı, 2019) and, in some cases, are accompanied by program staff during the implementation process (Grant and Francis, 2011; Brunzell et al., 2015a; Dorado et al., 2016; Washington State University, 2016, 2018; Wyatt et al., 2017; Demkowicz and Humphrey, 2019). The remaining 41.2% includes policies that may include informational materials for school staff and other stakeholders, as well as suggestions for their implementation in the school context, but do not include training or practical elements (Cole et al., 2005, 2013; Bloom, 2007; Brunzell et al., 2015b; Chafouleas et al., 2016; National Child Traumatic Stress Network Schools Committee (NCTSN), 2017; Alive and Well Community, 2019; Arora et al., 2021).

The implementation and realization of all concepts involve members of the school staff. In addition, implementation can involve collaborating entities, such as mental health services, as suggested in the concepts of Chafouleas et al. (2016), HTCL (Cole et al., 2005, 2013), TSSTP (Guarino and Chagnon, 2018), and implemented in HEARTS (Dorado et al., 2016), CLEAR (Washington State University, 2016), and HTL (Wolpow et al., 2009). In some cases, parents, usually those of the students in interest, are given the opportunity to attend training on trauma-sensitive approaches (Wolpow et al., 2009; Dorado et al., 2016). Among the programs, there are also some concepts in which the implementation process is accompanied by various offers by employees of these programs—often therapists or appropriately trained pedagogues—either as a fixed or an optional component of the concept (Bloom, 2007; Grant and Francis, 2011; Dorado et al., 2016; Washington State University, 2016; Wyatt et al., 2017; Demkowicz and Humphrey, 2019; Maya Vakfı, 2019). All of the above-mentioned concepts contain initial in-service training for school staff, supplemented by, for example, counseling (Bloom, 2007; Grant and Francis, 2011; Dorado et al., 2016; Washington State University, 2016; Wyatt et al., 2017) and supervision (Demkowicz and Humphrey, 2019). In addition, some programs offer the provision of therapy (Dorado et al., 2016; Maya Vakfı, 2019) or expand the program with the presence of project staff within the school (Dorado et al., 2016), as well as complementary offerings of training and/or materials (Bloom, 2007; Grant and Francis, 2011; Demkowicz and Humphrey, 2019). As described above, the American concepts account for the largest percentage of trauma-sensitive school concepts (58.8%). With the Sanctuary Model (Bloom, 2007), whose basic concepts were first transferred from TIC to the school concept before the turn of the millennium, and the HTCL (Cole et al., 2005, 2013), the oldest concepts are also available there. In the United States, the content of HTCL, in particular, has formed the basis for some of the more recent U.S. concepts, which have partially adopted and further developed elements of HTCL, including HEARTS (Dorado et al., 2016), TSSTP (Guarino and Chagnon, 2018), and HTL (Wolpow et al., 2009). Furthermore, the concepts behind HTL (Wolpow et al., 2009) and HEARTS (Dorado et al., 2016) were informed by the ARC framework (Kinniburgh et al., 2005) as well as CLEAR (Washington State University, 2016). The ARC framework is an approach to trauma-sensitive care that is transferred to concepts of trauma-sensitive preschools and kindergartens (Holmes et al., 2015); thus, it is only considered as a foundation for approaches based on it for the context of this review. The largest group within the American concepts are those that follow the rationale of MTSS. These concepts for trauma-sensitive schools, which were mostly developed from 2016 onwards, the tiered structure is almost identical (Chafouleas et al., 2016; Dorado et al., 2016; National Child Traumatic Stress Network Schools Committee (NCTSN), 2017; Guarino and Chagnon, 2018; Arora et al., 2021). In Australia, a total of four concepts of trauma-sensitive schools have been identified: BSEM (Brunzell et al., 2015a), TIPE (Brunzell et al., 2016), ReLATE (Diggins, 2021), and School’s In for Refugees (Grant and Francis, 2011). Globally, a trend of transferring U.S. concepts to other regions can be observed. In particular, the *Sanctuary Model* (Bloom, 2007) is mentioned as the basis for three of the four concepts identified from Australia (Brunzell et al., 2015a,b; Diggins, 2021). The BSEM (Brunzell et al., 2015a), in turn, was transferred to a school in Cambodia through an Australian organization and adapted to local needs (Wyatt et al., 2017). It has been adapted in six other countries, including Canada, Ireland, Mexico, Ecuador, Scotland, Israel, and Northern Ireland (Millen and MacDonald, 2012; Bunting et al., 2018). The authors of the ReLATE concept (Diggins, 2021) also report incorporating elements from the frameworks of Chafouleas et al. (2016), HTCL (Cole et al., 2005), and the National Child Traumatic Stress Network Schools Committee (NCTSN) (2017) into their concept. The Turkish Trauma Informed Schools concept (Maya Vakfı, 2019) also uses an MTSS structure that bears a strong resemblance to those of U.S. concepts (z. B. Guarino and Chagnon, 2018).

Three further concepts of trauma-sensitive schools were identified outside the United States and Australia, including one from England (Demkowicz and Humphrey, 2019), one from Turkey (Maya Vakfı, 2019), and one from Cambodia (Wyatt et al., 2017). In the United Kingdom, *Trauma Informed Schools UK* (TISUK) partners with various influential institutions, such as UNICEF, as well as various county governments and city councils to encourage schools across territories to participate in the programs. Beyond state borders, the organization provides training in Italy, China, and West Africa. The trainings are accompanied by supervision, conferences, and consultations for leaders (Demkowicz and Humphrey, 2019).

The *Trauma Informed Schools Program* (Maya Vakfı, 2019) is a collaborative project between the Istanbul-based Maya Vakfı organization and the United Kingdom-based *Theirworld* organization. In 2021, the program was awarded Qatar Foundation’s WISE Award 2021 (Qatar Foundation, 2021), which is given annually to six successful and innovative projects worldwide that address global education challenges. Despite the three-tiered structure, which bears resemblance to the structure of the U.S. MTSS models, no references to these concepts are made within the publications or website (Maya Vakfı, 2019). Following early evaluations, the Trauma Informed Schools program is receiving government support from the Turkish Ministry of Education, and it is being expanded from its current implementation in two provinces to nine provinces, with a recommendation to participate in the program currently under review by the Turkish government (Maya Vakfı, 2019; Theirworld, 2021).

The origin of the Hagar model can be found in the BSEM (Brunzell et al., 2015a), which was supplemented by various approaches from psychology and social work and adapted to specific regional needs (Wyatt et al., 2018). Due to missing evidence for a scientific foundation of the concept beyond these included approaches and on the components and implementation, studies are currently conducted to determine these elements (Wyatt et al., 2017, 2018).

### 3.2 Adaptation of content to the needs of students with a refugee background

At 82.4%, a majority of the concepts are designed to meet the needs of students with nonspecific causes of trauma, most commonly referred to as trauma resulting from ACEs. However, the concept of Arora et al. (2021) refers to students with a migrant background in adolescence, which explicitly includes young people with a refugee background and traumatization. The concept of Maya Vakfı (2019) focuses primarily on traumatized students who have fled from Syria to Turkey. The School’s In for Refugees (Grant and Francis, 2011) provides individualized concepts for schools serving traumatized refugee students. Accordingly, 17.6% of the identified concepts of trauma-sensitive schools contain specific measures for the group of traumatized students. In 35.3% of the concepts, traumatized students with a refugee background are explicitly mentioned as part of the target group (Cole et al., 2005; Bloom, 2007; Brunzell et al., 2015a; National Child Traumatic Stress Network Schools Committee (NCTSN), 2017; Guarino and Chagnon, 2018). 47.1% of concepts address trauma-related experiences that may occur among the group of traumatized students with a refugee background, although they are not explicitly listed as part of the target group (Brunzell et al., 2015b; Chafouleas et al., 2016; Dorado et al., 2016; Washington State University, 2016; Wyatt et al., 2017; Alive and Well Community, 2019; Demkowicz and Humphrey, 2019; Diggins, 2021).

Arora et al. (2021) designed their concept specifically for the group of potentially traumatized immigrant students in adolescence and, to adapt to this target group, focus on culturally sensitive interventions at all levels, family involvement, and implementation of interventions to treat trauma-related symptoms and other mental health problems in students who need such support. The concept of Maya Vakfı (2019) is specifically designed to meet the needs of refugee students from Syria who are educated in schools in Turkey. An adaptation to this group is present in program points of training for teachers, counselors, and school administrators that address concrete knowledge regarding trauma resulting from war and migration, loss and grief, and neglect and abuse. School’s In for Refugees (Grant and Francis, 2011) provides several resources to support school-wide planning and change processes, such as background information on refugees’ experiences and the impact of trauma on learning, development, and well-being. Furthermore, it provides case studies for school staff to gradually learn to appreciate the experiences of students with refugee backgrounds, to consider in the school context how trauma experienced by these children and youth can impact their learning, and to apply a whole-school approach to supporting them. The program’s website offers a comprehensive and freely accessible collection of materials with information, strategies at different school levels for elementary and secondary schools, downloadable materials, and workshops and training. The complete program and the individualized set of measures and materials for the school’s needs are free of charge, as the costs are fully covered by the State of Victoria (Australia).

### 3.3 Effectiveness of trauma-sensitive school concepts

A total of 12 studies were included in the evaluation of effectiveness, covering seven concepts (Table 2). One study is part of a dissertation (Delaney, 2020). Two studies are in a single document (Washington State University, 2016), so they are marked by a subscript number to distinguish them. Of the total, only three studies have been published in journals that include a peer-review process (Day et al., 2015; Dorado et al., 2016; Diggins, 2021). The remaining studies are freely available via the programs’ websites without quality assurance measures (Stokes and Turnbull, 2016; Washington State University, 2016, 2018; Farrelly et al., 2019; Maya Vakfı, 2019; Stokes et al., 2019; Delaney, 2020).

**Table 2:** Effects of trauma-sensitive school concepts.

Reference	Population	Intervention	Comparison	Outcome	Study Design
*Day et al. (2015)	*Participants:* Students: *n =* 70 *Specifics:* Age: 14–18 years (no mean value and standard deviation reported) Gender: only female Other specifics: all participants are court-involved School: *N* = 1 middle and high school of an institution for female students who are in court proceedings and have faced abuse or neglect in the past Country: USA	*Intervention:* Modified version of The Heart of Teaching and Learning: Compassion, Resiliency, and Academic Success (HTL; Wolpow et al., 2009) *Intervention components:* - School staff training: two half-day trainings and booster trainings occurring monthly over 2-h period *Implementation:* - Implementation period: eight months (October 2012–May 2013) - Control of Implementation fidelity: Classroom and teacher performance observations as well as individual coaching by a therapist certified in trauma and attachment	No comparison group	*Measured outcomes:* Student needs, post-traumatic symptoms, self-esteem, perceptions of school climate *Main results:* Significant reduction in post-traumatic symptoms with a low effect size (*d =* 0.30), no significant change in student needs, self-esteem, and school climate	*Study Design:* Pre–post design without a control group *Measures:* Student Needs Survey (Burns et al., 2006), The Child Report of Post-traumatic Symptoms (Greenwald and Rubin, 1999), The Rosenberg Self-Esteem Scale (RSE; Rosenberg, 1989), six close-ended questions developed by the research team to gather information on student perceptions of school climate *Analysis:* Paired-sample t-tests (pre- and post-test), calculation of effect sizes (Cohen’s *d*)
Delaney (2020)	*Participants:* Quantitative assessment: School staff (teachers, special needs assistants, the school principal, and the school psychologist): *n_1_* = 40; Qualitative assessment: School staff: *n_2_* = 14 participants from the intervention group *Specifics:* Age: No information reported Gender: No information reported School: *N* = 1 primary school Country: Ireland	*Intervention:* Trauma-Sensitive Schools Training Package (TSSTP; Guarino and Chagnon, 2018) *Intervention components:* - School staff training: three sessions (90 min) - Modules one and two of the TSSTP (“Understanding trauma and its impacts” and “Building trauma-sensitive schools”) *Implementation:* - Training period: two months (September–October 2019) - School staff training only, no implementation of trauma-sensitive practices	Waitlist control group School staff: *n_2_* = 19 (teachers and the school principal) School: *N* = 1 primary school	*Measured outcomes:* Knowledge and understanding of trauma and its impact on students, general self-efficacy and self-efficacy in dealing with traumatized students, staff perspective on their role in dealing with traumatized students, attitudes toward trauma-sensitive practices *Main results:* Significant increase in knowledge and understanding of trauma and its effects on students in the intervention group (g = 1.67 to 2.26 pre–post effect in the subscales), significant group effects (η^2^ = 0.30 to 0.49 in the subscales); significant increase in self-efficacy in dealing with traumatized students in the intervention group (g = 0.64 pre–post effect), significant group effect (η^2^ = 0.11 group effect post-intervention); significant increase in the teachers’ sense of efficacy in the intervention group (g = 0.46 pre–post effect), significant group effect (η^2^ = 0.09); no interaction effect between group and time or main effect for time and staff attitudes toward trauma-sensitive practices, no significant changes in the control group as well as staff perceptions of their role in dealing with traumatized students in either group	*Study Design:* 2×2 quasi-experimental, non-equivalent waitlist control group design, and sequential explanatory mixed-methods design *Measures:* The Teaching Traumatized Students Scale (Crosby et al., 2016), Knowledge and Understanding of Trauma and its Impact Assessment (Dorado et al., 2016), Staff Perception of Role Survey (Reker, 2016), Attitudes Related to Trauma-Informed Care-10 Item Form (Baker et al., 2016), Attitudes Related to Trauma-Informed Care-35 Item Form: Self-Efficacy Subscale Form (Baker et al., 2016), Teachers’ Sense of Efficacy Scale: Short Form (Tschannen-Moran and Hoy, 2001) *Analysis:* Pairwise comparisons and mixed between–within-subject ANOVAs, calculation of effect sizes (Hedges *g*; *η^2^*)
* Diggins (2021)	*Participants:* Students: *n* = 18 *Specifics:* Age: 9–16 years (mean: 12.5, SD: 1.95) Gender: 11% female, 89% male Other specifics: many with diagnoses in the areas of autism spectrum disorder, ADHD, or anxiety disorders School: *N* = 1 school (nongovernment alternate remedial school focusing on emotional and social development, P-12) Country: Australia	*Intervention:* Rethinking Learning and Teaching Environments (ReLATE; Diggins, 2021) *Intervention components:* - School staff training: two-day group training in the Sanctuary model (Yanosy et al., 2015); three-day group training in therapeutic crisis intervention - Schoolwide trauma-specific interventions - Community meetings (daily) - Safety plans - Therapeutic crisis intervention - Life space interviews - School staff debriefings to incidents - Supervisions - Clinical discussions with the psychologist (three times per term) *Implementation:* - Implementation period: 12 months (2019–2020) - Control of Implementation fidelity: no information	No comparison group	*Measured outcomes:* Emotional symptoms, behavioral problems, hyperactivity, peer problems, and prosocial behavior, impact of student’s behavior on family, home life, friendships, learning, and leisure activities, PTSD symptoms *Main results:* Over 12 months: significant decrease in scores for conduct problems (*d* = 0.88), peer problems (*d* = 0.40), and total social difficulties, prosocial skills (*d* = 0.35); a decrease in emotional symptoms and hyperactivity (*d* = 0.72) did not reach significance; effect sizes are larger after 12 months than after six months; parents report positive effects of the concept on the home environment, friendships, learning, and leisure activities	*Study Design:* Mixed-methods design (pre–post follow-up assessment without a control group and interviews) *Measures:* Parent report from Strengths and Difficulties Questionnaire (SDQ; Goodman, 2001), PTSD Checklist (PCL-PR; Blanchard et al., 1996) *Analysis:* Analyses of variance (ANOVAs), calculation of the reliable change indicator and effect sizes (Cohen’s *d*)
*Dorado et al. (2016)	*Participants:* School staff (teachers, principals, social workers, special educators, counselors): n_1_ = 175; Students n_2_ = 1,243, including 67 students who received adjunctive therapy through HEARTS *Specifics:* Age: No information was reported for the total samples Gender: 47% female, 63% male Schools: N = 4 HEARTS schools (three elementary schools, one school with kindergarten through 8th grade) Country: USA	*Intervention:* Healthy Environments and Response to Trauma in Schools (HEARTS; Dorado et al., 2016) *Intervention components:* - MTSS - School staff training and consultation - Schoolwide trauma-specific interventions (Tiers 1–3) - Trauma-specific, culturally congruent therapy for trauma-impacted students by HEARTS clinicians (Tier 3) *Implementation:* - Implementation period: school A: five continuous years, school B: four years with short interruptions, school C: two years, school D: one and a half years (2009–2014) - Control of Implementation fidelity: no information	No comparison group	*Measured outcomes:* School staff: knowledge about trauma and its effects on children, understanding how to help traumatized children learn in school, knowledge about trauma-sensitive practices, knowledge about burnout and vicarious traumatization, use of trauma-sensitive practices; Students: ability to learn, time on task in the classroom, time spent in the classroom, school attendance, number of disciplinary office referrals and suspensions over time, and clinical and psychosocial needs and strengths *Main results:* School staff: significant increase in perceived knowledge and its effect on children (*d* = 1.72), understanding of how to help traumatized children learn in school (*d* = 1.56), knowledge about trauma-sensitive practices (*d* = 1.67), knowledge about burnout and vicarious traumatization (*d* = 1.43) and use of trauma-sensitive practices (*d* = 1.28) Students: significant increase in students’ ability to learn (*d* = 0.89), time on task in the classroom (*d* = 0.86), time spent in the classroom (*d* = 1.00), and school attendance (*d* = 0.54); Reduction in total negative incidents by 32% at one year and 87% at five years (*d* = 2.42)	*Study Design:* Quantitative retrospective pre–post assessment *Measures:* HEARTS Evaluation Survey (Dorado et al., 2016), Child and Adolescent Needs and Strengths scale (CANS; Dorado et al., 2016) *Analysis:* Within-subjects paired t-tests (pre- and post-test), calculation of effect sizes (Cohen’s *d*)
Farrelly et al. (2019)	*Participants:* School staff: *n_1_* = 4; Students: *n_2_* = 7; Darebin Community Renewal Officer: *n_3_* = 1; Berry Street trainers: *n_4_* = 2 *Specifics:* Age: No information reported Gender: No information reported School: *N* = 2 primary school Country: Australia	*Intervention:* Berry Street Education Model (BSEM; Brunzell et al., 2015a) *Intervention components:* - School staff training: four days over two years - Three tiers of therapeutic learning: repairing the student’s regulatory abilities (Tier 1), repairing the student’s disrupted attachments (Tier 2), and increasing the psychological resources (Tier 3) *Implementation:* - Implementation period one to two years (2017–2019) - Control of Implementation fidelity: high implementation fidelity while adapting strategies to contextual needs	No comparison group	*Measured outcomes:* School staff: teachers’ teaching practices, school-wide practices; Students: student well-being, engagement, and achievement *Main results:* School staff: use of new classroom strategies, teacher confidence, and well-being; Positive effects on teacher understanding of student behavior, improved communication, and relationships between teachers and students; Students: no significant effects on student well-being, engagement, and achievement	*Study Design:* Qualitative design *Measures:* Individual and focus group interviews *Analysis:* No information reported
Maya Vakfı (2019)	*Participants:* Quantitative assessment: School staff (teachers and school counselors): *n_1_* = 63; Qualitative assessment: School staff: *n_2_* = 7 teachers from the intervention group *Specifics:* Age: 21–59 years (mean: 39.18, SD: 10.43) Gender: 74% female, 27% male School: *N* = 4 primary schools Country: Turkey	*Intervention:* Trauma-Informed Schools (Maya Vakfı, 2019) *Intervention components* - MTSS - School staff training: two modules over a 6-h training period - School counselor training: two modules over - Schoolwide trauma-specific interventions (Tiers 1–3) - Individual therapy sessions in the Maya Vakfı field office (Tier 3) *Implementation:* - Training period: No information reported - School staff training only, no implementation of trauma-sensitive practices	No comparison group	*Measured outcomes:* Beliefs and knowledge of trauma and child abuse *Main results:* Significant increase in beliefs and knowledge; perceived increased level of awareness and sensitivity in understanding trauma	*Study Design:* Mixed-methods design (quantitative pre–post assessment and interviews) *Measures:* Self-developed scales, semi-structured in-depth interviews *Analysis:* Paired-sample t-tests (pre- and post-test)
*Stokes (2022)	*Participants:* Quantitative assessment: School staff (leadership, teachers, educational support staff): *n_1_* = 35 (2019); *n_1_* = 30 (2020); *n_1_* = 34 (2021); Students: *n_2_* = 192 (2019); *n_2_* = 256 (2020); *n_2_* = 260 (2021); Qualitative assessment: School staff (leadership, teachers, educational support staff): *n_3_* = 12; Students: *n_4_* = 20 *Specifics:* Age (students): 7–12 years (no mean value and standard deviation reported) Gender: No information reported School: *N* = 1 school (low socio-economic index) Country: Australia	*Intervention:* Trauma Informed Positive Education (TIPE; Brunzell et al., 2016) *Intervention components* - School staff training: four whole days and further master classes - Coaching program for teachers - Development of a trauma-informed instructional model by the school leadership - Implementation of TIPE strategies in the classroom - Non-punitive behavior management system *Implementation:* - Implementation period: one and a half years (2019–2021); the study is part of a larger four-year longitudinal study - Control of Implementation fidelity: implementation guided by the TIPE trainer	No comparison group	*Measured outcomes:* School staff: Understanding of trauma and its impact on students, effective teaching methods, learning environment, collaboration of school staff in school planning; Students: student attitudes to school, student behavior *Main results:* School staff: greater understanding of trauma and its impact on students by the school staff, individualization of TIPE strategies for their school, increase in perceived collaboration among school staff in school planning, and a more positive perceived learning environment after three years; Students: fewer punishments, positive changes in school policies and instructional practices that support their learning, improvements in student-teacher relationships, and an improvement in social interaction	*Study Design:* Mixed-methods design (quantitative pre–post assessment and interviews) *Measures:* School Staff Survey (Victorian State Government Department of Education and Training, 2021), Student Attitudes to School Survey (Victorian State Government Department of Education and Training, 2022); in-depth interviews *Analysis:* Interview analysis using the framework of Miles and Huberman (1994); total scores for quantitative measures
Stokes et al. (2019)	*Participants:* Quantitative assessment: Students: *n_1_* = 911; Qualitative assessment: School staff and training staff (principals, assistant principals, BSEM leaders, well-being leaders): *n_2_* = 17; Students: *n_3_* = 51 *Specifics:* Age (students): years 5–9 (no specific age, mean value and standard deviation reported) Gender: No information reported Schools: *N* = 3 (two primary schools, one P-9 school, low socio-economic index) Country: Australia	*Intervention:* BSEM (Brunzell et al., 2015a) *Intervention components* - School staff training: four whole days and further master classes - Design of a developmental curriculum Focused on Five domains: Body, relationship, stamina, engagement, and character - Implementation of classroom strategies from the BSEM curriculum - On-going professional development and advice by the Berry Street training team - Train-the-trainer model *Implementation:* - Implementation period: three years (2015–2017) - Control of Implementation fidelity (part of the research question): different implementations of the concept at the three schools with some commonalities	Three schools with the same intervention	*Measured outcomes:* School staff: training effectiveness, understanding of trauma and its impact on students, implementation of the BSEM, teacher practice, social relationships; Students: understanding and use of BSEM strategies, social relationships, psychological functioning, student attitudes to school, critical incidents and suspension, school attendance *Main results:* School staff: greater understanding of trauma and its impact on students by the school staff, identifying students’ triggers, support students to regulate their behavior, positive impact on student-teacher and peer relationships; in interviews, teachers report changes in their teaching practice by providing a BSEM toolkit of activities and strategies, improving their ability to regulate themselves in dealing with difficult situations; Students: positive changes in self-perception, behavioral regulation, and peer and teacher-student relationships over time and across all schools; in interviews, students report that BSEM has provided them with helpful strategies to shape their relationships, behavior, and learning,	*Study Design:* Mixed-methods design (quantitative measurements two times per year); focus group interviews *Measures:* Self-report online survey for students (not specified); Student Attitudes to School Survey (Victorian State Government Department of Education and Training, 2022); focus group interviews with individual representatives of all groups *Analysis:* No information was provided for the analysis of interviews, descriptive analysis of quantitative data
Stokes and Turnbull (2016)	*Participants:* Quantitative assessment: Students: *n_total_* = 2050; *n_1_* = 150 (school 1), *n_2_* = 615 (school 2, intervention group), *n_3_* = 1,285 (school 2, control group); Qualitative assessment: School staff (teachers and school leadership): *n_3_* = 9 (school 1), *n_4_* = 19 (school 2), *n_5_* = 26 (school 1); Students: *n_6_* = 26 (school 2) *Specifics:* Age (students): years 5–8 (no specific age, mean value and standard deviation reported) Gender: No information reported Schools: *N* = 2 (one primary school, one P-9) Country: Australia	*Intervention:* BSEM (Brunzell et al., 2015a) *Intervention components* - School staff training: sequence of professional development workshops, seminars, training sessions, and follow-up sessions - Design of a developmental curriculum focused on five domains: body, relationship, stamina, engagement, and character - Implementation of classroom strategies from the BSEM curriculum *Implementation:* - Implementation period: one year (2014–2015) - Control of Implementation fidelity: different implementations of the concept at the two schools (whole school vs. one area)	Two schools with the same intervention, school 2 split into an intervention and a control group (control group: *n_3_* = 1,285)	*Measured outcomes:* Student well-being, student achievement, student engagement, student attitudes to school, critical incidents, and suspension *Main results:* Improvement in student wellbeing, achievement, student engagement, and attitudes to school decrease in suspensions and critical incidents	*Study Design:* Mixed-methods design (quantitative pre–post assessment with control group); focus group interviews *Measures:* Student Attitudes to School Survey (Victorian State Government Department of Education and Training, 2022); focus group interviews *Analysis:* No information was provided for the analysis of interviews, descriptive analysis of quantitative data
Washington State University (2016)	*Participants:* *n_total_* = 11,651 students (*n_1_* = 2,585 in intervention schools, *n_2_* = 9,065 in comparison schools) *Specifics:* Age: Years 3–5 (no specific age, mean value and standard deviation reported) Gender: No information reported Schools: *N_1_* = 6 intervention schools, *N_2_ =* 20 comparison schools without CLEAR interventions Country: USA	*Intervention:* Collaborative Learning for Educational Achievement (CLEAR; Washington State University, 2016) *Intervention components* - MTSS - School staff training: three-year progressive training process; cumulative 1-h trainings: nine trainings in year 1, six trainings in year 2, four trainings in year 3 - Progressive elaboration of best-practice trauma principles - Whole-school actions and instructional practices to improve learning outcomes - Individual or small group consultation support, participation in the monthly professional development (PD) trainings *Implementation:* - Implementation period: one year (2014–2015) - No information reported regarding control of Implementation fidelity: No information reported	Three independently selected matched comparison groups of schools without CLEAR intervention	*Measured outcomes:* School performance in English language and math *Main results:* English Arts Standardized Test: significant increase in English language proficiency for the CLEAR intervention group, with the percentage of tests passed increase for the intervention group and no change for the control group. Math State Test: average increase of two percentage points in the intervention group, consistent with slightly decreased average percentage points in the control group	*Study Design:* Pre–post design with three control groups *Measures:* English Arts Standardized Test, Math State Test *Analysis:* Repeated measures analyses of covariance
Washington State University (2016)	*Participants:* School staff: *n =* 432 *Specifics:* Age: No information reported Gender: No information reported School: *N =* 12 (10 elementary schools, one middle school, and one high school) Country: USA	*Intervention:* CLEAR (Washington State University, 2016) *Intervention components* - MTSS - School staff training: three-year progressive training process; cumulative one-hour trainings: nine trainings in year 1, six trainings in year 2, four trainings in year 3 - Progressive elaboration of best-practice trauma principles - Whole-school actions and instructional practices to improve learning outcomes - Individual or small group consultation support, participation in the monthly professional development (PD) trainings *Implementation:* - Implementation period: one year (44% of the sample), two years (27% of the sample), three years (29% of the sample) - Control of Implementation fidelity: significant variation across participating schools in the level of reported integration of the six CLEAR practices, significant variation across staff	No comparison group	*Measured outcomes:* Implementation of CLEAR principles, impact of CLEAR on their practice, school climate; student behavior, student–teacher engagement, shift in school policies and practices, predictors of change *Main results:* Significant increase in all areas of CLEAR principles, significant increases in the implementation of TIC methods and school characteristics, significant increases in the areas of school climate, student behavior, and staff–student collaboration; effects often stronger the longer CLEAR was implemented	*Study Design:* Pre–post design without a control group (retrospective baseline reporting strategy) *Measures:* Web-based survey to assess the implementation of the CLEAR principles *Analysis:* Repeated-measure ANOVAs with implementation year (first, second, or third program year), linear regression analysis of predictors of change in practice or perception of school characteristics
Washington State University (2018)	*Participants:* School staff: *n =* 432 *Specifics:* Age: No information reported Gender: No information reported Schools: *N =* 13 (13 elementary schools) Country: USA	*Intervention:* CLEAR (Washington State University, 2016) *Intervention components* - MTSS - School staff training: three-year progressive training process; cumulative 1-h trainings: nine trainings in year 1, nine trainings in year 2, six trainings in year 3 - Progressive elaboration of best-practice trauma principles - Whole-school actions and instructional practices to improve learning outcomes - Individual or small group consultation supports, participation in the monthly professional development (PD) trainings *Implementation:* - Implementation period: nine schools with an implementation period of one year (2017–2018), three schools with an implementation period of three years - Control of Implementation fidelity: different quality of implementing conditions across communities	No comparison group	*Measured outcomes:* Implementation of CLEAR principles, impact of CLEAR on their practice, school climate; student behavior, student–teacher engagement, shift in school policies and practices, predictors of change *Main results:* Significant increase in all variables related to school staff, stronger effects in all areas after three years of implementation than after one year; no significant effects related to physical safety for students and school staff and respectful behavior on the part of students	*Study Design:* Pre–post design without control group (retrospective baseline reporting strategy) *Measures:* Web-based survey to assess the implementation of the CLEAR principles *Analysis:* No information reported

Asterisks mark a publication in peer-reviewed journals.

About half of the studies cover evaluations in schools in the United States. Notably, five studies examined the effects of trauma-sensitive schools in Australia (Stokes and Turnbull, 2016; Farrelly et al., 2019; Stokes et al., 2019; Diggins, 2021; Stokes, 2022) and one each in Ireland (Delaney, 2020) and Turkey (Maya Vakfı, 2019). Both primary and secondary schools are represented in the samples. While the setting of the other studies included regular schools, the surveys in Day et al.’s (2015) and Diggins’s (2021) study were conducted in special settings. The sample sizes of the quantitative surveys sometimes show large differences, with a minimum of *n* = 18 students (Diggins, 2021) and a maximum of *n* = 11,651 students (Washington State University, 2016). In addition, unlike all other studies that examined members of the school staff and, in some cases, students, Diggins (2021) conducted a parent survey.

Within studies, a variety of study designs are used, including mixed-methods designs (Stokes and Turnbull, 2016; Maya Vakfı, 2019; Stokes et al., 2019; Delaney, 2020; Diggins, 2021; Stokes, 2022), quantitative pre–post surveys (Day et al., 2015; Dorado et al., 2016; Washington State University, 2016, 2018), of which two studies used non-randomized control groups. The Washington State University study used three control group clusters, and the Delaney (2020) study used an asymmetric waitlist control group design. In addition, Farrelly et al. (2019) have chosen a qualitative design. Within the mixed-methods surveys and the isolated quantitative surveys, there are sometimes major methodological differences, especially regarding the sample size and the only partially standardized test procedures used to collect the data.

The independent variables naturally vary about the concepts evaluated. Three studies have examined the impact of CLEAR (Washington State University, 2016, 2018). Data on the effects of the concept were collected in a study one year after implementation of the concept (Washington State University, 2016). In each of the two other studies, data are collected in schools at different intervals from the time of implementation (Washington State University, 2016; Washington State University, 2018). In one study (Day et al., 2015), the effects of using HTL (Wolpow et al., 2009) on court-involved female students who have faced abuse or neglect in the past are examined. This is a modified version of the concept, supplemented by two additional interventions. The time interval between the implementation of the concept and the survey is eight months. Dorado et al. (2016) assessed the effects of the HEARTS program in four different schools where the concept was implemented for varying periods of time, ranging from one and a half to five continuous years. In most of the studies, no indication has been reported regarding the realization of the concepts in schools. In the studies regarding BSEM (Stokes and Turnbull, 2016; Farrelly et al., 2019; Stokes et al., 2019; Stokes, 2022), the schools’ implementation of the concept is part of the surveys, so it is presented as results. In two studies, only the impact of training on teachers and the effect of implementing the concept were examined (Maya Vakfı, 2019; Delaney, 2020).

The outcome variables examined can be divided into four groups that examine the effects of the intervention (implementation of the concept or participation in training) on school personnel, school- and/or classroom-level aspects, student-related dimensions, and trauma-related symptoms. Positive effects (see Table 2) have been reported at the student level in behavioral variables (Dorado et al., 2016; Stokes and Turnbull, 2016; Washington State University, 2016, 2018; Stokes et al., 2019; Diggins, 2021; Stokes, 2022), dimensions of well-being and (Stokes and Turnbull, 2016; Farrelly et al., 2019) relationship variables (Stokes et al., 2019; Stokes, 2022), as well as school performance (Washington State University, 2016). While all of these variables represent potential indicators of positive effects on sublevels of trauma-sensitive school concepts, they provide little insight into their comprehensive impact on the various dimensions of student impairment in the school setting and their complex interactions. The same applies to the reported results with regard to the school and class level. In terms of impact at the faculty level, findings related primarily to self-perceived implementation and use of trauma-sensitive practices (Dorado et al., 2016; Stokes and Turnbull, 2016; Washington State University, 2016, 2018; Farrelly et al., 2019; Stokes et al., 2019; Stokes, 2022), changes related to self-perceived knowledge of trauma-related issues and self-perceived skills related to appropriate handling and teaching of traumatized students (Dorado et al., 2016; Washington State University, 2018; Maya Vakfı, 2019; Delaney, 2020), and attitudes toward trauma-sensitive schools (Maya Vakfı, 2019; Delaney, 2020).

Since the two studies by Delaney (2020) and Maya Vakfı (2019) only conducted the trainings of the programs but did not implement the concepts and collect their effects, only effects regarding school staff can be taken from them. Delaney’s (2020) dissertation reports significant increases in knowledge and understanding of trauma and its impact on students, perceived self-efficacy concerning this group, and attitudes regarding trauma-sensitive practices in the intervention group, with no change in the control group. Increases in teachers’ self-perceived knowledge and skills are also reported in Maya Vakfı’s (2019) study, but these values do not reach significance. None of the available studies evaluated the effects of trauma-sensitive school concepts on traumatized students with refugee backgrounds.

## 4 Discussion

Internationally, 17 concepts of trauma-sensitive schools meet the inclusion criteria of this review. Only a few of the existing concepts primarily refer to the target group of traumatized students with a refugee background. In 35.3% of the concepts, they are explicitly included in the target group, while in 47.1% of the concepts, they are not named as a target group. Three of the concepts available at the time of the research include specific measures for traumatized students with a refugee background (Grant and Francis, 2011; Maya Vakfı, 2019; Arora et al., 2021). Referring to the concept of Arora et al. (2021), it must be stated in a limiting way that traumatized students with a refugee background are only listed as a subgroup of adolescents with a migration background in the United States. Due to the drastic differences in migration history and the often associated increased exposure rate of children and adolescents with a refugee background to traumatizing events (Wood et al., 2020), it can be assumed that those student’s needs regarding trauma-sensitive school concepts might be different from those of students with a migration background but without a refugee background. In addition to the three concepts mentioned above, six of the 17 concepts explicitly mention students with a refugee background as part of their target group. In eight cases, they were not explicitly mentioned in the concept descriptions. This deficit of concepts with specific adaptations to the group of traumatized students with a refugee background can be explained by the fact that trauma-sensitive concepts are still a comparatively recent development (Cohen and Barron, 2021); thus, concepts are initially developed and established with an unspecific target group but can be flexibly adapted to the individual starting situations and needs in schools.

Given the immense diversity of potentially traumatic experiences of children and adolescents with a refugee background (Wood et al., 2020), it can be assumed that concepts that address the needs of students with ACEs, in general, may nevertheless have intersections regarding the needs of traumatized children and adolescents with a refugee background. Therefore, it is possible that traumatized students with a refugee background can also benefit from concepts of trauma-sensitive schools that are primarily aimed at students with ACEs. Some concepts, such as the TLPI’s *flexible framework* (Cole et al., 2005, 2013), explicitly pointed out that the underlying concept is to be seen only as an orientation framework for the individual design of a trauma-sensitive school, whose concrete implementation is based on the individual needs of the student body and the conditions at the school. Accordingly, the frameworks have the inherent potential to be adapted to the specific and individual needs of traumatized students with a refugee background. The three approaches that take this subgroup into account include special cultural sensitivity, training of school staff and providing information on trauma resulting from war and migration (Maya Vakfı, 2019), as well as the experiences of refugees and building an appreciative attitude toward them (Grant and Francis, 2011) specifically for supporting refugee students in trauma-sensitive approaches (Arora et al., 2021). These elements can also be found in some concepts without explicit reference to students with a refugee background, these or similar elements can also be found. For example, the element of cultural sensitivity is included in the core principles of Substance Abuse and Mental Health Services Administration (SAMHSA) (2014) or transferred to the school context in the concept of Chafouleas et al. (2016), in the TSSTP (Guarino and Chagnon, 2018), in HEARTS (Dorado et al., 2016), and in the concept of National Child Traumatic Stress Network Schools Committee (NCTSN) (2017). Therefore, it cannot be discounted that these concepts implicitly include adaptations to the group of traumatized students with a refugee background, without explicitly mentioning them in the context of the present descriptions.

Concerning their worldwide distribution, the greatest diversity of concepts of trauma-sensitive schools is found in the United States, while several concepts are found in Australia and isolated concepts in Turkey, the United Kingdom, and Cambodia. The results of this study indicate that trauma-sensitive school concepts are largely developed and implemented in countries with high financial resources. Exceptions are the two concepts from Turkey and Cambodia, whose development was supported by organizations based in the United Kingdom and Australia, respectively. While these are also among the largest third host countries, a high number of children and adolescents with refugee backgrounds seek protection primarily in countries with low financial resources, including many African countries (United Nations High Commissioner for Refugees, 2022), which is why it can be assumed that concepts of trauma-sensitive schools sometimes do not reach the places where they are most needed under the current conditions.

Empirical studies on the effectiveness of trauma-sensitive school concepts are not available for all concepts and show considerable differences in terms of their research designs and data collection methods, as well as low significance. Furthermore, no concepts were identified for which effectiveness regarding students with a refugee background was reported. In terms of the effects of implementing trauma-sensitive school concepts in general, the study focused on the impact of the training and support provided by the programs, which in and of themselves provide few clues about the positive effects of trauma-sensitive school approaches on students or the various actors in the school context. For instance, increased knowledge and positive attitudes can potentially have an impact on changes in teaching practice (Baumert and Kunter, 2006). However, the studies do not contain any information about a concrete implementation of these aspects and the effect of this changed teaching practice.

In addition to these difficulties of comparability, many of the existing studies show deficiencies concerning their methodological quality; often, no control groups are included. Currently, there are only a few studies on the needs of the heterogeneous group of students who have experienced trauma resulting from their experience of flight concerning the school context and on the knowledge and competencies that teachers must have to be able to adequately support these students. There is a need for further research to develop high-quality teacher training that enables teachers to implement trauma-sensitive concepts in schools and to establish them in the long term. Altogether, it can be stated that the development of trauma-sensitive schools is still in its infancy (Simon et al., 2020; Cohen and Barron, 2021). The future spread of trauma-sensitive school concepts on a global level is currently difficult to estimate. Due to growing global migration movements, the need will undoubtedly also grow concerning children and adolescents with a refugee background.

The establishment of trauma-sensitive concepts in schools underlines the importance of teachers as social caregivers (Popham et al., 2023), and it emphasizes their importance in supporting social integration and, thus, the psychosocial development of students (Boda et al., 2023). Social integration supports refugee students’ well-being, psychosocial development, and academic success (Stadtfeld et al., 2019). Conversely, poor social integration constitutes a risk factor for these outcomes (Wolke et al., 2013). Given that refugee students often experience poor social integration and lack friendly peer relationships (Boda et al., 2023), promoting social integration should be viewed not only as a supportive but also as a mandatory component in teacher behavior. Social support not only affects direct trauma-related issues but is also significantly correlated with behavioral, emotional, and cognitive engagement in school, which are considered crucial determinants of students’ educational success (Wang and Eccles, 2012, 2013). In this context, schools are social organizations that offer the potential to promote social integration and the closely related social–emotional learning of students in a systematic way (Eccles and Roeser, 2011).

Concerning the methodological limits of the present study, it must be noted that false negatives cannot be ruled out due to the methodological approach and the restrictions concerning access to the content of possible further trauma-sensitive school concepts. On the one hand, this relates to the selection strategy when searching via Google Scholar, where after 500 results under the algorithm preset by the search platform for sorting by relevance, a content saturation of the search results was observed, as a result of which the titles were screened with less care. On the other hand, non-English-language concepts, if present, were not considered due to the selection strategy. This also applies to articles with regional access restrictions and commercialized programs whose content can only be accessed after paying a fee. This has a particularly limiting effect on the results for questions one to three.

The research and selection were conducted by a single person. Although the involvement of a second scientist in the literature search can certainly help to ensure the reliability and completeness of the search and minimize possible bias, this was not done here, as the search was conducted in a highly standardized manner. The results of the present study provide an important insight into trauma-sensitive school concepts available worldwide with a focus on the special needs of refugee students. Above all, they show how these concepts should be developed and empirically evaluated in an evidence-based manner.

There is an increasing number of children and young people on the run, which additionally implies a considerable need for research into the effects of refugee-related traumatic experiences of students as well as their mechanisms of action within families and the resulting needs. This is elementary to be able to respond effectively and in a targeted manner to the educational and socio-political challenges associated with the inclusion of traumatized students with a refugee background.

## Data availability statement

The original contributions presented in the study are included in the article; further inquiries can be directed to the corresponding author.

## Author contributions

EL: Writing – original draft, Writing – review & editing. FL: Project administration, Supervision, Writing – review & editing. GC: Project administration, Writing – review & editing.
